# Telemonitoring and Protocolized Case Management for Hypertensive Community-Dwelling Seniors With Diabetes: Protocol of the TECHNOMED Randomized Controlled Trial

**DOI:** 10.2196/resprot.5775

**Published:** 2016-06-24

**Authors:** Raj Padwal, Finlay Aleck McAlister, Peter William Wood, Pierre Boulanger, Miriam Fradette, Scott Klarenbach, Alun L Edwards, Jayna M Holroyd-Leduc, Kannayiram Alagiakrishnan, Doreen Rabi, Sumit Ranjan Majumdar

**Affiliations:** ^1^ Department of Medicine University of Alberta Edmonton, AB Canada; ^2^ Department of Computing Science University of Alberta Edmonton, AB Canada; ^3^ Department of Medicine University of Calgary Calgary, AB Canada

**Keywords:** blood pressure, hypertension, seniors, telemonitoring, randomized controlled trial, case management

## Abstract

**Background:**

Diabetes and hypertension are devastating, deadly, and costly conditions that are very common in seniors. Controlling hypertension in seniors with diabetes dramatically reduces hypertension-related complications. However, blood pressure (BP) must be lowered carefully because seniors are also susceptible to low BP and attendant harms. Achieving “optimal BP control” (ie, avoiding both undertreatment and overtreatment) is the ultimate therapeutic goal in such patients. Regular BP monitoring is required to achieve this goal. BP monitoring at home is cheap, convenient, widely used, and guideline endorsed. However, major barriers prevent proper use. These may be overcome through use of BP telemonitoring—the secure teletransmission of BP readings to a health portal, where BP data are summarized for provider and patient use, with or without protocolized case management.

**Objective:**

To examine the incremental effectiveness, safety, cost-effectiveness, usability, and acceptability of home BP telemonitoring, used with or without protocolized case management, compared with “enhanced usual care” in community-dwelling seniors with diabetes and hypertension.

**Methods:**

A 300-patient, 3-arm, pragmatic randomized controlled trial with blinded outcome ascertainment will be performed in seniors with diabetes and hypertension living independently in seniors’ residences in greater Edmonton. Consenting patients will be randomized to usual care, home BP telemonitoring alone, or home BP telemonitoring plus protocolized pharmacist case management. Usual care subjects will receive a home BP monitor but neither they nor their providers will have access to teletransmitted data. In both telemonitored arms, providers will receive telemonitored BP data summaries. In the case management arm, pharmacist case managers will be responsible for reviewing teletransmitted data and initiating guideline-concordant and protocolized changes in BP management.

**Results:**

Outcomes will be ascertained at 6 and 12 months. Within-study-arm change scores will be calculated and compared between study arms. These include: (1) clinical outcomes: proportion of subjects with a mean 24-hour ambulatory systolic BP in the optimal range (110-129 mmHg in patients 65-79 years and 110-139 mmHg in those ≥80 years: primary outcome); additional ambulatory and home BP outcomes; A1c and lipid profile; medications, cognition, health care use, cardiovascular events, and mortality. (2) Safety outcomes: number of serious episodes of hypotension, syncope, falls, and electrolyte disturbances (requiring third party assistance or medical attention). (3) Humanistic outcomes: quality of life, satisfaction, and medication adherence. (4) Economic outcomes: incremental costs, incremental cost-utility, and cost per mmHg change in BP of telemonitoring ± case management compared with usual care (health payor and societal perspectives). (5) Intervention usability and acceptability to patients and providers.

**Conclusion:**

The potential benefits of telemonitoring remain largely unstudied and unproven in seniors. This trial will comprehensively assess the impact of home BP telemonitoring across a range of outcomes. Results will inform the value of implementing home-based telemonitoring within supportive living residences in Canada.

**Trial Registration:**

Clinicaltrials.gov NCT02721667; https://clinicaltrials.gov/ct2/show/NCT02721667 (Archived by Webcite at http://www.webcitation.org/6i8tB20Mc)

## Introduction

### Impact of Hypertension in Seniors With Diabetes

Diabetes is present in more than 20% of seniors (defined herein as age ≥ 65 years) and often leads to devastating complications and premature death. Hypertension affects over 80% of seniors with diabetes and is widely viewed as the most important cause of cardiovascular complications and death in these patients. Despite its critical importance to health, hypertension remains undertreated and uncontrolled in approximately 40% of seniors with diabetes [1].

Aggressive blood pressure (BP) reduction substantially reduces mortality, cardiovascular events, and microvascular complications in all patients with diabetes [2]. Seniors are at particularly high risk for hypertension-related complications and derive greater treatment benefit than younger patients (ie, greater absolute risk reduction) [3,4]. Achieving BP control in high-risk patients, including those with diabetes, is cost saving (which is rare, as few medical interventions save money over the long term) [5]. Contemporary Canadian guidelines recommend a treatment target BP ≤130 mmHg for these individuals; however, 52% of Canadian seniors with diabetes do not achieve this target [1,6]. Treatment consists of health behavior modification (low sodium diet, optimizing weight, exercise) and antihypertensive drugs [6]. Angiotensin-converting enzyme inhibitors or angiotensin receptor blockers are first-line agents, dihydropyridine calcium channel blockers second line, and thiazide diuretics third line [6,7]. Of note, most patients with diabetes and hypertension will need multiple medications to achieve adequate BP control [8].

### Treatment of Hypertension in Seniors With Diabetes: A Complex Care Challenge

The need for aggressive BP control in seniors with diabetes must be balanced against the very real risk of serious treatment-related complications. Seniors, especially those who have cognitive impairment or who are frail, very old, or institutionalized, are more likely to experience treatment-related complications. These include hypotension, postural dizziness, syncope, falls, and metabolic side effects (including high/low potassium, sodium, and to a lesser extent, elevated glucose levels) [9-12]. Autonomic dysfunction and postural hypotension, more common in older individuals and in those with diabetes, may limit uptitration of antihypertensive drugs even if sitting BP levels are above target [10]. Dose reduction or drug discontinuation may be warranted in many patients to avoid inappropriate polypharmacy, adverse effects (plus additional drug treatments that are used to treat these adverse effects), need for laboratory monitoring, and costs.

Antihypertensive dose reduction or drug discontinuation is clearly warranted when serious adverse effects manifest. However, in asymptomatic patients with low BP, no obvious trigger is present to signal the need for dosage reduction. Furthermore, no widely accepted threshold definition for low BP exists at which dose reduction/discontinuation is mandated. In our opinion, systolic BP (SBP) levels <110 mmHg may confer increased risk for hypotension (and <100 mmHg clearly increase risk) [13].

Importantly, there is no randomized trial evidence that clinical benefits occur from reducing BP to <110 mmHg in seniors with diabetes [9]. For this reason and because it is critically important to avoid drug-related adverse effects (which are quite common [11]), reducing therapy when SBP is below 110 mmHg seems warranted unless a compelling nonhypertension-related indication for an antihypertensive agent is present. The extent to which dosage reductions are made in asymptomatic seniors in real-world clinical practice is unclear. We speculate that dosage adjustments are rarely made either because patients are not monitored frequently (thus, the low BP is not detected) or because a low BP fails to trigger an appropriate dose correction (because providers are primarily trained to focus on high not low readings). This might change if monitoring and a protocol that triggered appropriate dosage modification were implemented.

BP management in seniors with diabetes is further complicated by current guidelines that permit higher SBP treatment targets (<150 mmHg versus the usual <130 mmHg) in patients aged ≥80 years [6,14]. The intent of these guidelines is to allow practitioners to use more lenient targets in seniors who are frail or who, based on clinical opinion, may not tolerate lower BP levels. These guidelines allow us to define an upper limit to the BP therapeutic range for most seniors, above which more aggressive antihypertensive therapy to lower BP should be considered.

### Types of BP Monitoring

To ensure that BP levels are neither too high nor too low, accurate BP monitoring is required. Serial office BP measurements are currently used to monitor the vast majority of Canadians with hypertension. Unfortunately, office readings are inaccurate frequently because recommended measurement techniques are not followed or equipment is not regularly calibrated [15]. Furthermore, in seniors, office measurements are falsely high (white coat effect) in 15%-20% of cases and falsely low (masked effect) in 10%-15% of cases [16]. An additional disadvantage of office measurements is that patients are required to attend clinical appointments, a barrier for seniors who don’t drive or who have mobility or financial limitations. Office measurements, therefore, may be infrequent, and this limits the ability to make timely therapeutic adjustments to address low or high BP.

Because of these limitations of office BP monitoring, contemporary guidelines strongly endorse use of out-of-office measurement [6]. Out-of-office measurement has additional advantage over office BP in that it allows multiple temporally separated readings to be performed. This provides a more accurate assessment of true BP because BP is a continuous parameter that changes every second of the day. Out-of-office measurement is currently performed by measuring 24-hour ambulatory BP monitoring (ABPM) or home BP monitoring. ABPM is widely regarded as the gold-standard measurement method [6,17], but is not widely available, not widely reimbursed, and often not well tolerated (because frequent measurements are needed and sleep disturbance may occur). Home BP measurement is thus much more commonly used for follow-up BP measurement [6]. Nearly 50% of hypertensive Canadians own a home monitor [18]. Home measurement has additional advantages, it increases treatment adherence and patient activation (by encouraging BP self-monitoring) [19-21], and when used alone, modestly reduces SBP (by 1.3 mmHg [95% CI 0.3-2.2] in a meta-analysis of 17 studies) [22].

### Methods of Performing Home BP Measurement

Measuring a home BP series is the recommended method of performing home BP measurement [6]. A home BP series is composed of duplicate readings in the morning and evening (ie, 4 per day) daily for 7 days [6]. Readings taken on the first day are discarded and the latter 6 days (24 measurements) are averaged. If BP levels are at target, the home BP series is repeated quarterly. If BP levels are uncontrolled, therapeutic adjustments are made and the home BP series repeated in 4 weeks.

Home readings can be used in 1 of 3 major ways: (1) by the patient alone (who bears responsibility for giving the readings to their provider); (2) via telemonitoring, in which readings are automatically summarized and sent to the care provider; and (3) through telemonitoring plus protocolized case management, in which the summarized readings are reviewed by a case manager authorized to adjust treatments. BP telemonitoring ± case management is not being used in Canada because data on effectiveness and feasibility in this country are limited, the required technological infrastructure is not available, and a provider reimbursement plan does not exist.

Although contemporary guidelines strongly endorse home BP measurement, describe how to self-measure BP and outline how to perform a home BP series, more needs to be done to ensure correct uptake in clinical practice [6]. A major drawback is the onus is placed on the patient to measure, record, and present the home readings to their care provider though, this patient alone method has the advantage of requiring no additional resources and is the predominant method used in Canada. However, patients often forget to record their measurements, do not follow the recommended protocol (timing, frequency, and number of measurements), and/or self-select readings for presentation to their physician [23,24]. Recent data indicate that less than one-third of patients report ≥80% of measurements to their physician [25]. Important physician-related barriers to proper use of home BP measurement also exist. Physicians often do not calculate the mean BP (treatment adjustments are based on the mean), do not scan and upload hand-written BPs into their Electronic Medical Record (thus, no permanent record is available), and/or do not act on out-of-target readings (“therapeutic inertia”) [23,24].

Home BP telemonitoring is a second method that, through process automation and protocols, can potentially overcome some of the aforementioned barriers [25,26]. BP telemonitoring consists of electronically and securely transmitting remotely collected BP measurements in real time to a central electronic health care portal. Data can be summarized for use by patients and providers, this includes calculation of BP means and graphing temporal trends in BP. Mean BPs that are too high or low can be flagged for action, whereas those in the normal range provide evidence for optimal control. Telemonitoring may eliminate the need for in-person clinic visits, and contributes to health care delivery efficiency and making better use of provider time. A recent meta-analysis of 23 randomized controlled trial (RCTs; 7037 patients) reported that home BP telemonitoring reduced BP by 5/3 mmHg compared with usual care (*P*<.0001 for both SBP and diastolic BP) [27]. This is a clinically important reduction, a 5-mmHg reduction in BP in high-risk patients (including diabetes) reduces cardiovascular events by 15% [28] and, in patients with diabetes, reduces stroke by 13% [29].

The third method of implementing home BP monitoring is to combine telemonitoring with case management. Case managers, usually nurses or pharmacists, work collaboratively with patients and physicians to optimize health behaviors, monitor risk factors, implement therapeutic adjustments, encourage adherence, and coordinate follow-up care [30-32]. Case management is well established and is currently used in contemporary clinical practice (our Pharmacare industry partner specializes in providing pharmacist case management services to seniors living in apartments, lodges, and assisted living facilities). Case management works best when the case managers have prescribing authority and use algorithms or protocols to make guideline-concordant therapeutic initiations and adjustments [30,33,34]. This can potentially overcome therapeutic inertia. BP improvements are greater when interventions have combined case management with telemonitoring [27]. Thus, it is essential to study case management because it may be needed in conjunction with telemonitoring to maximize the effectiveness of the latter.

### Reasons Why Home BP Telemonitoring is not Currently Used in Canada

Collaboration between health care providers, decision makers, and device makers/technology companies is limited. This collaboration is required to make telemonitoring feasible. Historically, such collaborations have been rare, primarily because of lack of dialogue and interaction among potential partners.

Canadian data are very limited. A 1-year study in 110 hypertensive patients with diabetes (age ≥30 years; mean age 63 years) home BP telemonitoring and automated cellphone text messages that instructed patients to seek follow-up care was compared with usual care [35]. BP was reduced by 7.1/2.3 mmHg (*P*<.005) in the telemonitoring arm, and there was a 20% increase in the proportion of patients with controlled BP (51% versus 31%; *P*<.05). To our knowledge, this is the only published Canadian study of relevance. Patients did not receive treatment recommendations, seniors were not specifically studied, cost-effectiveness was not assessed, usability or acceptability not reported, and case management was not used. For these reasons, another trial is warranted to build on this important foundational work.

Costs have, historically, been a major barrier. This is primarily because of uncertainty over who will pay for teletransmission, health portal development, and portal maintenance. However, home BP monitors are now inexpensive and widely used, cellphone or Internet use is very high (enabling convenient, secure electronic data transmission), payment solely for teletransmission is not necessary (ie, as long as an existing data plan is present, the extra data usage for intermittent BP teletransmission is minimal), and established companies exist that specialize in health data transmission and health portal creation and maintenance. Thus, because of these technological advancements, fewer barriers remain. Importantly, BP control in high-risk populations (including diabetes) is cost saving [5]; therefore, health care payors funding or subsidizing this expenditure can expect initial costs to be offset by substantial downstream savings. In the United States, health care payors can spend an additional $600-1250 USD per patient per year controlling BP in high-risk patients yet still remain cost-neutral, this is a huge “safety margin” that supports the potential for telemonitoring to be cost-effective (because total costs are likely to be under these thresholds). Because previous studies have demonstrated mixed results in terms of BP telemonitoring cost-effectiveness [27,36], it is important to create a system that minimizes costs, maximizes cost-effectiveness, and leverages expenditures already borne by the individual for other reasons (ie, mobile phones, set top boxes, and data plans) to promote health care system sustainability.

Need for user training had been nearly prohibitive. This has largely been eliminated through technological advancements and major advances in user-friendliness. Systems require little additional action (other than BP self-measurement) because BP teletransmission can be automated once the reading is taken.

### Summary of Rationale for a Tech-Based Canadian Study in High-Risk Seniors

To summarize, hypertension is very common in seniors with diabetes and substantially increases morbidity, mortality, and health costs. Controlling BP markedly reduces complications and can be cost saving. However, BP reduction is not the only goal, in some cases (white coat effect, low BP), drug dosage reductions are appropriate. BP management in seniors with diabetes is complicated by the need to balance cardiovascular risk reduction against the risk of adverse effects and polypharmacy. Age-appropriate BP thresholds and targets in the very elderly (age ≥ 80 years) must also be considered. Effective BP management is further hindered by the near ubiquitous dependence on inconvenient, infrequently performed and inaccurate office BPs to titrate therapy. Home BP readings should be used instead, but the optimal implementation method in terms of effectiveness, acceptability, and costs remains unclear. Measuring and reporting home BP could be left up to the patient, automated using telemonitoring, or automated and protocolized using telemonitoring and case management.

Although over 20 published trials reported clinically important BP reductions using home BP telemonitoring and case management, data in seniors are lacking, and it is important to confirm feasibility, effectiveness, safety, usability, and acceptability in this population in Canada. Importantly, prior studies have focused on reducing high BP only; in seniors, avoiding low BP and polypharmacy are equally important. Telemonitoring has initial (BP device) and ongoing (teletransmission and health portal maintenance) costs. Case management costs must also be considered. These costs ought to be offset by cost reductions achieved through avoidance of hypertension-related complications, drug-related adverse events, and reduced drug use. If done the way we propose, telemonitoring has the potential to be highly cost effective in these high-risk patients. However, a formal economic analysis is needed before widespread implementation can be justified.

### Objectives

This Telemonitoring and Protocolized Case Management for Hypertensive Community-Dwelling Seniors With Diabetes (TECHNOMED) trial is designed to (1) assess the “real world” effectiveness and safety of home BP telemonitoring alone or in combination with protocolized pharmacist case management in seniors with diabetes and hypertension when compared with “enhanced” usual care; (2) evaluate the usability and acceptability of home BP telemonitoring; and (3) examine the cost-effectiveness of home BP telemonitoring alone and home BP telemonitoring plus protocolized case management.

In aggregate, these objectives will assess the impact on a comprehensive range of outcomes important to patients, providers, decision makers, industry partners, and funders.

## Methods

### Study Design

**Figure 1:**
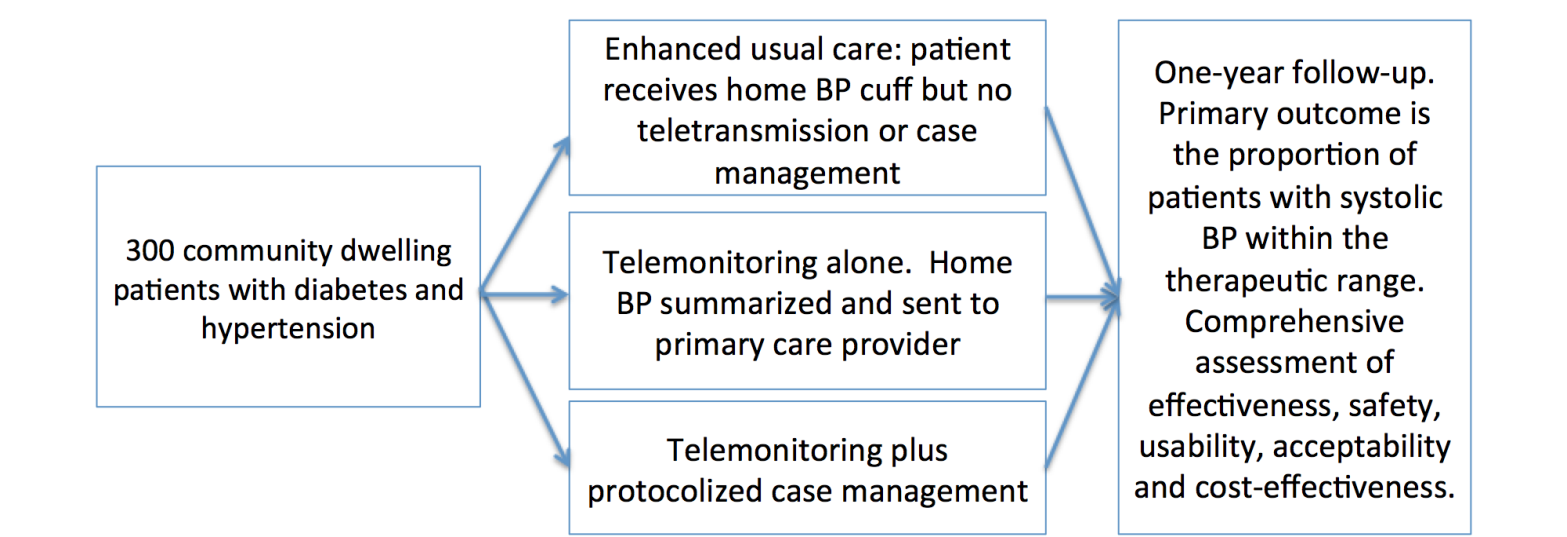
Study design.

In this 1-year pragmatic, prospective randomized open label trial with blinded ascertainment of end points, 300 patients will be randomly assigned (1:1:1) to one of 3 study arms (Figure 1): (1) enhanced usual care (in which participants will be given a home monitor but BP teletransmission will not be accessible and case management not available to them); (2) home BP telemonitoring alone; and (3) home BP telemonitoring plus protocolized case management.

### Randomization

Computer-generated randomization will be performed centrally and independently by the EPICORE center (www.epicore.ualberta.ca) to ensure allocation concealment from all research personnel. Randomization will be stratified by baseline SBP (<140 mmHg versus ≥140 mmHg). Although clinic staff and pharmacist case managers cannot be blinded to allocation status, all outcome assessments will be performed by research assistants working independently from regular clinic staff and the pharmacist case managers.

### Recruitment

Consecutive, consenting seniors (aged ≥65 years) will be recruited from seniors independent living or supportive living residences in greater Edmonton.

#### Inclusion Criteria (All Criteria Must Be Met)

These include: (1) age ≥65 years with a documented diagnosis of diabetes and hypertension, and (2) adequate English fluency, both verbal and written.

#### Exclusion Criteria (Any 1 Sufficient to Exclude)

These include: (1) SBP level >220 mmHg or diastolic BP level >110 mmHg on screening BP measurement (WatchBP [Microlife Corp., Widnau, Switzerland]); (2) heart failure; (3) severe cognitive impairment, defined as a score of ≥5 on the Short Portable Mental Status Questionnaire [37]; (4) severe depression (Patient Health Questionnaire [PHQ-8] ≥15) [38]; (5) foreshortened life expectancy (<1 years); (6) participation in a concurrent cardiovascular trial; and (7) currently receiving case management services for cardiovascular risk factor control.

### Telemonitoring Intervention

The telemonitoring system will be built in collaboration with TeleMED (www.telemeddiagnostic.com). TeleMED, a Canadian company specializing in the electronic management of noninvasive diagnostic test data, will provide their services in-kind. All patients will receive a validated electronic upper arm oscillometric BP device (A&D Ltd. UA-651BLE; San Jose, CA) and a set top box that will enable wireless transmission of BP readings. This equipment will remain in their residence for the duration of the study. All patients will be shown how to view their BP readings on their device. Pushing a single button activates the device and initiates a BP measurement, which is autotransmitted to the set top box via a Bluetooth low-energy connection. Once set top box receives the data, it encodes the data to prevent “sniffing” by a third party. Without any further action required by the patient, the data are sent to a dedicated research server. The server decodes the data and encrypts it using the Advanced Encryption Standard with 256-bit key and inserts the encrypted data into the database. The research server is physically located at the University of Alberta in a secure facility accessible only to authorized personnel. The data are then securely pushed to TeleMED, where it is summarized in a web portal for provider use.

Patients will be instructed to perform all measurements according to recommended techniques for home BP measurement (Table 1). Four measurements will be taken daily for 1 week. If BP is uncontrolled (high or low), this 1-week of measurements will be done each month until BP is in the therapeutic range. Once controlled, the 1-week protocol will be repeated every 3 months, as recommended by contemporary guidelines [6]. Teletransmitted BP readings will be summarized within the health portal and an overall weekly mean will be calculated (first-day measurements will be discarded and the subsequent 24 measurements taken over the next 6 days will be averaged) [6]. This mean will be used for clinical management decisions. Temporal trends will be plotted to graphically summarize the data for provider use.

**Table 1:** Blood pressure measurement.

Method	Details
24-hour ambulatory BP^a^monitoring	BP readings will be taken every 15 minutes during the daytime and every 30 minutes at night with a Spacelabs 90227 device. Twenty-one readings during the daytime and 7 during nighttime will be required for a successful study [ 39]. Otherwise, a repeat study will be necessary. Patients will be given a diary to record the times that they retire to and arise from bed. Day and night intervals will be defined according to these patient-reported times and used to determine the daytime and nighttime BP averages. Timing of drug administration will also be recorded. Patients will also be instructed to go about their daily activity but refrain from exercising for the duration of the monitoring period and to stand still with their arm at their side when the monitor cuff is inflating.
Home BP measurement	Two measurements 1 minute apart will be taken in the morning between 0800 and 1000 and 2 measurements will be taken in the evening between 1800 and 2200 taken using the A&D home device. This will be done on 7 consecutive days for 1 week. If BP is uncontrolled (high or low), this 1-week measurement protocol will be repeated each month until BP is within the therapeutic range. Once controlled, the 1-week protocol will be repeated every 3 months.
Automated office BP	Three reading average in both arms taken while seated plus 1 supine reading and 1 standing reading taken at 1 min and 3 min using the WatchBP device.

^a^BP: blood pressure

**Figure 2:**
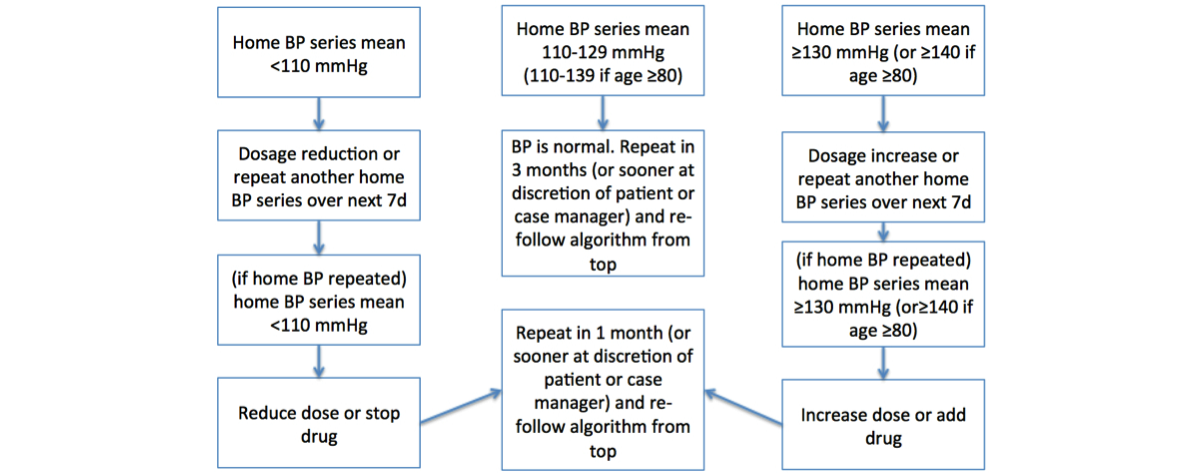
Case manager protocol for antihypertensive drug titration.

**Figure 3:**
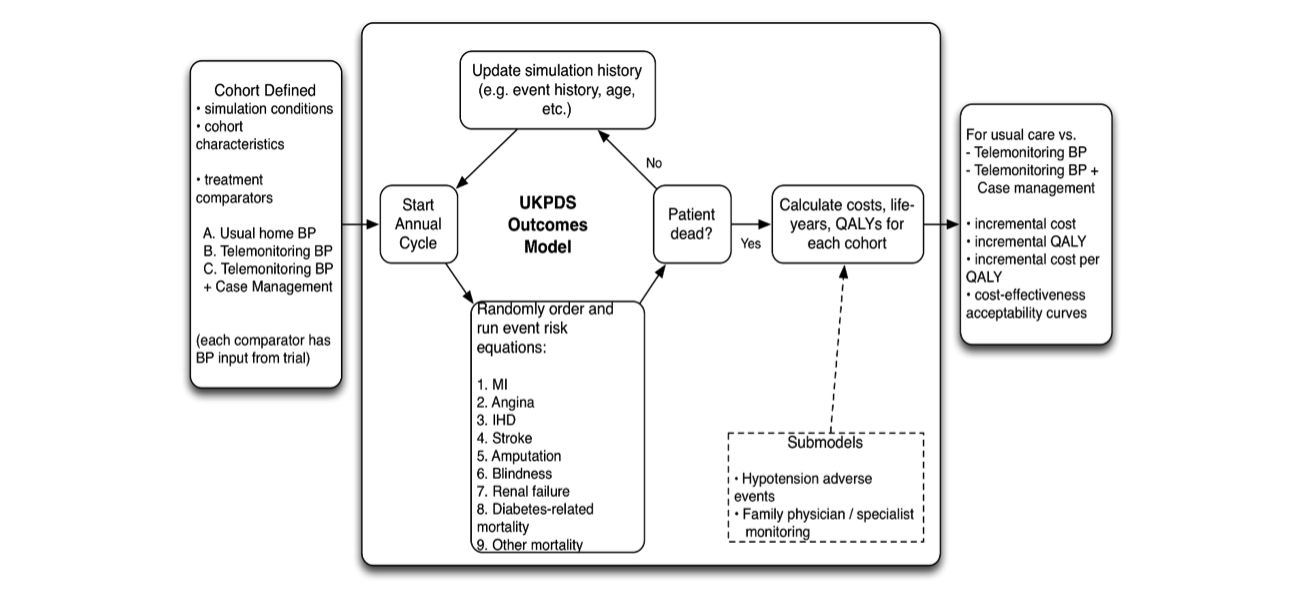
Overview of economic model.

BP teletransmission will occur in all study arms but will be used differently in each:

- Enhanced usual care: Home BP readings will be teletransmitted for data collection purposes but neither patients nor providers will have access to the teletransmitted readings. High BP levels that trigger safety alerts to research personnel are the only exception, patients and their primary care providers will be made aware of these (see safety end points below). This is nevertheless considered “enhanced” usual care because patients receive a home BP monitor, are taught how to measure home BP, and are encouraged to take BP readings to appointments with their providers. In addition, they will be reminded to perform a home BP series each quarter for study outcome purposes, which will encourage self-monitoring. This reflects contemporary Canadian recommendations [6]. A summary of the Canadian hypertension guidelines will also be faxed to each primary care provider at the time of patient enrolment [6]. Recognizing that it takes years for guideline adoption to occur, we suspect that many patients in this arm will be managed solely using office BP measurements despite the potential availability of patient-reported (but not teletransmitted) home BP readings.
- Telemonitoring alone: Home BP series mean, trends, and individual readings will be faxed to the primary care provider with a 1-page summary of Canadian guidelines for BP thresholds, targets, and treatments [6].
- Telemonitoring plus protocolized case management: Patients in this arm will each be assigned a pharmacist case manager who holds full prescribing privileges and who will (1) administer health behavior modification counselling, teach BP self-monitoring, and monitor medication adherence; (2) review telemonitored health portal BP summaries and make protocolized therapeutic adjustments if appropriate (Figure 2); (3) fax a summary of these adjustments to the participant’s primary care provider (to make them aware of treatment changes); and (4) facilitate communication between patients and providers.

Pharmacare (www.mypharmacare.ca), an Alberta company specializing in pharmaceutical service delivery, will provide pharmacist case managers as an in-kind contribution to the study. Pharmacare case managers hold full drug prescribing licences, enabling them to independently initiate and titrate drugs. To ensure full guideline concordant standardization of the intervention, case managers will undergo a training session with a group of clinical experts from the University of Alberta Hypertension Clinic (who also serve on the executive of Hypertension Canada) on home BP monitoring and hypertension guidelines before study initiation.

Medication regimen adjustments will be performed according to a guideline-concordant protocol [6]. Drugs will be added in the following order: angiotensin-converting enzyme inhibitor or angiotensin receptor blocker, dihydropyridine calcium channel blocker, thiazide diuretic, beta-blocker, spironolactone, doxazosin, clonidine, and hydralazine. Drugs will be reduced or stopped in reverse order. When initiating new agents, the longest-acting agents in each class will be used.

### Data Collection Including End Points

Unless otherwise indicated, data will be collected at baseline, 6 and 12 months after randomization. Study personnel will collect data using standardized case report forms. Home BP series data will then be sent to secure servers housed within the Department of MedIT, Faculty of Medicine and Dentistry, University of Alberta and then forwarded to the Web portal.

Baseline data collection will include(1) demographics and health behaviours: age, sex, race, marital status, smoking, and alcohol intake; (2) past medical history: atrial fibrillation, dyslipidemia, coronary artery disease, stroke or transient ischemic attack, peripheral vascular disease, chronic kidney disease (glomerular filtration rate ≤60 mL/min and/or proteinuria), syncope, bradyarrhythmias, pacemaker, and history of hyponatremia, hyperkalemia, or hypokalemia; (3) past diabetes-related information: duration of disease, presence of retinopathy, neuropathy, nephropathy, amputation, symptomatic hypoglycemia, and use of insulin; (4) medication history including antihypertensive drugs: name, type, dosage, and frequency. This will be based on self-report and then will be cross-indexed with the patient’s pharmacy medication record; (5) anthropomorphic indices: height, weight, body mass index, and waist circumference; (6) upper mid arm circumference (to determine proper cuff size): measured using a tape measure half way between the acromion and the olecranon with the arm at heart level; (7) 24-hr ambulatory BP, home BP series, and pulse rate: BP will be measured according to recommended techniques (Table 2) using validated devices [40]. Three screening BP measurements will be taken while seated in each arm at baseline with the validated WatchBP office automated device to determine if exclusion criteria are present [41]. Lying and standing BP at 1 and 3 minutes will also be taken. A 24-hr ambulatory BP and mean 24-hour heart rate will be measured using the validated Spacelabs 90227 monitor (Snoqualmie, Wash) [42]. A home BP series taken with the UA-651BLE oscillometric home device (A&D Ltd., San Jose, CA) will be performed as outlined (Table 2). (8) Laboratory investigations will include serum sodium, potassium and creatinine; glycated hemoglobin (A1c); lipids (total cholesterol, high-density or HDL cholesterol, low-density or LDL cholesterol, triglycerides), urinary albumin or creatinine ratio and electrocardiogram; (9) Montreal Cognitive Assessment Scale **:**validated cognitive assessment instrument [43]; (10) Clinical Frailty Score: a validated 9-point instrument, with frailty defined as a score of 5 or more [44]; (11) health care use in past year includes physician visits, emergency department use, and hospitalizations ascertained through patient self-report and by linking to provincial administrative data sources and the provincial electronic health record; (12) quality of life and utility measurement: assessed using the EQ-5D [45]; (13) depression/anxiety measured using the Patient Health Questionnaire (PHQ-8) for depression [38] and the Generalized Anxiety Disorder Scale (GAD-2) for anxiety [46] These end points are being evaluated to ensure that increased monitoring does not lead to greater depression or anxiety via adoption of the “sick role” [35]; and (14) satisfaction with medical care: assessed similar to other studies that we have conducted [47-49] using the validated Patient Satisfaction Questionnaire (PSQ) [50], scored on a 5-point Likert Scale.

**Table 2:** Costing data for economic analysis.

Identification	Measurement	Valuation	Comments	
**Program Development**				
Health care professional time (physician, nurse, pharmacist)	Estimated hours for each health care professional to create care algorithm (algorithm start-up costs); Estimated hours for staff training to administer care algorithm (training costs)	Alberta Health Services wage rates, Alberta Health Care Insurance Plan/Alternate Funding Plan	Cost per patient estimated by plausible number of patients in program/managed per staff	
IT infrastructure	Equipment required to setup BP^a^telemonitoring and hours of IT support will be estimated from local experience and TeleMED input.	Wage rates. Price lists of IT equipment from manufacturer.	Costs apportioned over # of patients monitored in region over 5 years (estimated lifespan of equipment).	
**Program Delivery**				
Equipment	Number of home BP cuffs (standard and telemonitoring)	List price	Includes expected lifetime/repair costs and replacement.	
Internet/data	Mobile phone device/data plan used for telemonitoring (tested in sensitivity analysis).	Local cost of lowest priced suitable service	Included in sensitivity analysis, may be paid by health provider or patient (societal perspective)	
Medication use	Type, dose, frequency, and duration of use.	Alberta Blue Cross		
Health care professional time (pharmacist)	Estimated hours for staff to administer care algorithm (ongoing costs)	Alberta Health Services wage rates, Alberta Health Care Insurance Plan/lternate Funding Plan	Cost per patient estimated by plausible number of patients in program/managed per staff	
Staff costs/infrastructure	IT support/telemedicine portal/fax costs	TeleMED	Costs apportioned over # of patients monitored in region over 5 years.	
**Utilization**
Physician visits	Number of primary care or hypertension specialist visits over 12 months (patient reported).	Alberta Health Ambulatory Care Case Costing (utilizing National Ambulatory Care Reporting System)	Telemonitoring ± case management may reduce need for physician visits for BP management, scenarios tested in SA.	
Emergency department visits	Number of emergency room visits over 12 months attributable to BP or complications of treatment	Alberta Schedule of Benefits	Study may be underpowered to detect emergency department visits. Safety end points will be examined and likely resource use for each safety end point will be estimated.	
Hospitalizations	Number of hospitalizations over 12 months attributable to BP or complications of treatment	Alberta Health administrative data		
Societal costs	Patient and caregiver time costs for physician visits, emergency department visits, out of pocket medication costs. May also include data/mobile phone costs (explored in sensitivity analysis).	Standard Alberta wage rates (human capital approach)	Explored using a societal perspective.	

^a^BP: blood pressure

The 6- and 12-month follow-up data (measured as described previously) will be (1) clinical end points and patient-centered outcomes (as described previously): 24-hr ABPM, automated BP (seated, lying, and standing as described previously), telemonitored home BP, heart rate, medications, anthropomorphics, cardiovascular risk factors and markers (A1c, lipids, smoking, urinary albumin), cognition, frailty score, health care use (physician visits, emergency department visits and hospitalizations ascertained through self-report and via linked administrative health care data), quality of life and utilities, satisfaction with medical care, and depression and anxiety. (2) safety end points includes the frequency of (a) nonmechanical falls, syncope, hypotension requiring third-party assistance or medical attention and (b) electrolyte disturbances (hypokalemia [<3.3 mmol/L], hyperkalemia [>5.0 mmol/L], and hyponatremia [<130 mmol/L]. In addition, potentially life-threatening adverse effects will trigger an immediate alert causing the study team to notify the Data and Safety Monitoring Board, which will act independent of the study team to contact the patient and arrange appropriate nonstudy medical follow-up. Triggers for DSMB follow-up will include a BP ≥220/110 mmHg or a SBP <70 mmHg; potassium level ≤2.7 or ≥5.5 mmol/L; sodium level ≤126 or ≥152 mmol/L; or a PHQ-8 score ≥15, indicating severe depression. (3) User acceptability: will consist primarily of qualitative data collection. In addition, two 10-point Likert scales evaluating usability and acceptability will be collected. (4) Costing data: costing will adhere to the three-step microcosting technique of identification, measurement, and valuation of relevant health care and non–health care resources [51,52] and are outlined below and in Table 2. Resource use by category, including program start-up costs and on-going costs for each study arm, will be tabulated. In a 10% random sample of patients, time-motion studies related to the case manager will be conducted. The cost per patient will be calculated, and where any uncertainty in resource use or costs exists, plausible ranges of resource use will be determined and tested in sensitivity analysis. Resource use and cost data will be used to determine the overall and per-patient total costs, and incremental costs of interventions (telemonitoring ± case management) compared with usual care will be calculated [51,52].

### Analytic Plan for Major Outcomes

#### Aim 1: Effectiveness of Telemonitoring ± Protocolized Case Management

All primary analyses will be conducted according to the intention-to-treat principle. The primary outcome is the 1-year change in proportion of patients with overall 24-hour SBP in the optimal range (SBP is used because it is a stronger predictor of risk and because diastolic BP is rarely elevated in seniors [10]). We will use 24-hour ABPM because it is the gold-standard measurement method and the best validated clinical trial BP end point [40]. The 24-hr ABPM therapeutic range will be 110-129 mmHg in patients aged 65-79 years and 110-139 mmHg in those ≥ 80 years. Justification for the upper thresholds chosen is based on current guidelines that specify an overall 24-hour ABPM of ≥130 mmHg as high in all patients, including those with diabetes [6]. In patients ≥80 years, we will allow the option of a higher 24-hour target of <140 mmHg (ambulatory BP threshold definitions of normal versus high are lower than office BP thresholds; therefore, this threshold is analogous to the Canadian guideline concordant <150 mmHg office BP target that is allowed in older frailer patients [6]).

Major secondary outcomes will include the change in mean 24-hour SBP and diastolic BP (overall, daytime, and nighttime). Home BP and the automated BP measurements taken at each study visit will be examined similarly. Additional major outcomes will include postural BP changes and changes in A1c, lipids, anthropomorphic indices, quality of life, depression/anxiety, satisfaction with medical care, resource utilization, and the safety end points described previously.

##### Data Analysis

First, variables will be examined descriptively and graphically, including assessments of temporal trends and tests of normality. Second, the 6-month and 1-year mean change from baseline in each outcome will be calculated and compared between study arms (each intervention arm to the control arm, then between intervention arms) using chi-square tests for dichotomous outcomes and unpaired *t*-tests for continuous outcomes. Third, multivariable predictors of the 1-year change in a given outcome will be identified using appropriately constructed and calibrated logistic regression models for dichotomous outcomes (including the primary outcome) or linear regression models for continuous ones. Initial models will adjust for age, sex, SBP, and residential site (eg, Greater Edmonton Foundation versus Rosedale). Examples of additional covariates that may be examined include sociodemographic variables, comorbidities, baseline medications, primary care physician, and tests of potential interaction.

##### Sample Size Considerations

The study will be adequately powered to detect a clinically important 20% absolute difference in the primary outcome between each intervention arm and usual care and similar 20% difference between the 2 intervention arms (20% has been previously identified by a consensus of Canadian experts as the required minimum clinically important difference for any new hypertension [or any other cardiometabolic] intervention directed at patients with diabetes [53]). Based on pilot data collected in 60 seniors residing in supportive living at two Edmonton sites, only 18% were within the therapeutic range at baseline. Assuming 5% improvement with usual care (related to secular or temporal trends and trial participation or Hawthorne type effects), 20% further improvement with telemonitoring, and a further 20% improvement with telemonitoring + case management, a 2-tailed alpha of 0.05, power of 0.80, the required sample size will be ~80 patients per arm or 240 total. Accounting for ≈20% attrition over 1 year, 100 patients per arm or 300 patients total will be recruited.

#### Aim 2: Usability and Acceptability of Telemonitoring

Assessment of usability and acceptability is critical for all technology-enhanced care interventions because unanticipated and undesired effects can commonly occur after implementation [54-56]. End-user input into system design and operation is needed throughout the evaluation process; otherwise, interventions risk being ineffective, unusable, or unsafe [54,55]. Usability testing involves assessment of the human-computer interaction and, specifically, issues related to use, interface, design, and function are examined [57,58]. System evaluation is performed iteratively and includes assessment then redesign and retesting.

Usability and acceptability testing (device, data transmission, health portal) will be performed using well-accepted frameworks [57,59-61]. The evaluation will focus on functional goals (features, format, and interface), usability needs (outcome impact goals, end users’ requirements, and information needs), and end-users’ perceptions of the facilitators and barriers to use. End users that will be considered will include random samples of seniors with hypertension, family members who are primary caregivers, pharmacist case managers, and primary care physicians. We will perform usability and acceptability testing of the refined telemonitoring intervention at the beginning of the study and during the study. The evaluation will use standard mixed methods approach, with focus groups of all stakeholders, semistructured indepth interviews with think aloud and talk back with patients, and repeated surveys regarding the technology itself [54,55,57,58,60,62,63].

#### Aim 3: Cost-Effectiveness of Telemonitoring ± Case Management

##### Outcomes

The total and incremental cost for each intervention compared to usual care will be calculated (Table 2 and Figure 3), and the cost per decrement in SBP (cost-effectiveness model A), and incremental cost/QALY gained (cost-utility model B), will be determined.

##### Methods

A validated economic model (used by the research team in prior work examining interventions in type 2 diabetes [64,65]) will be modified and used to compare both telemonitoring alone and telemonitoring + case management versus usual care (Figure 3), adhering to recommended best practices for conduct of economic evaluation [51,66]. The patient population simulated will have characteristics of the patients studied (seniors with diabetes and hypertension). The model will be informed by primary data from the RCT, including detailed costing data (Table 2), utility scores (EQ-5D), and BP changes for each treatment strategy. A 1-year time horizon and a public health care payer perspective will be used for model A, where the surrogate of change in BP will be examined (cost per mm of reduction in SBP). No discounting of costs and benefits will be performed in the reference case (given the short time frame). We will also perform a cost-utility analysis (model B) linking the validated surrogate of BP reduction at 1 year to longer term health outcomes (including probability of developing heart disease, stroke, kidney failure, blindness) [67,68], and associated increased risk of death, decrement in quality of life, and increased health care costs with these events. The model will also incorporate other study end points including avoidance of low BP and reductions in adverse effects that result in health or resource use (short-term reduction in quality of life, physician, or emergency room visits), and other health care utilization that may be impacted by treatment strategy (cost of BP drugs, clinic visits to monitor and manage BP, and hospitalizations to treat adverse effects).

We have previously used the United Kingdom Prospective Diabetes Study (UKPDS) outcomes model to examine the cost-effectiveness of interventions in patients with type 2 diabetes [64,65]. Advantages of this model include robust validation [69], ability to specify characteristics of the patient population, and previous adaptation of the model to represent the Canadian context for resource use, costs, and quality of life [64,65]. Uncertainty and variability will be explored through sensitivity analysis, including one-way and probabilistic sensitivity analysis including cost-effectiveness acceptability curves, where a range of willingness to pay thresholds are examined. Sensitivity analysis considering a range of estimates obtained from the RCT (for example, 95% CI in BP differences) as well as other plausible ranges of parameters will be performed. These include assignment of some costs to either patient or health care payer (device, set top box, data plan, health portal access fee) or a range of costs of delivering telemonitoring ± case management for varying economies of scale.

### Subgroup Analyses and Substudies

Analyses of interest include examination of the effect of telemonitoring in subjects aged 80 years or greater as well as substudies on vascular stiffness, orthostatic changes, and novel BP measurement methods. In addition, passive, long-term follow-up using linked administrative data are planned to ascertain effects on cardiovascular morbidity and mortality.

### Ethics, Funding, and Registration

All subjects will provide written informed consent. The TECHNOMED trial protocol has been approved by the University of Alberta Research Ethics Board (PRO00051624), and the trial has received peer reviewed funding from the Canadian Institutes of Health Research (grant #EH2-143571) and Alberta Innovates Health Solutions (grant #201900506). The trial has been formally registered at clinicaltrials.gov (NCT02721667).

## Discussion

In summary, the TECHNOMED trial is a pragmatic randomized control trial that will comprehensively study, in the Canadian context, home BP telemonitoring in seniors. It will compare 3 different methods of implementing home BP measurement and examine a broad range of outcomes important to patients, providers, caregivers, and policy makers.

Home BP telemonitoring has been shown to effectively reduce BP and improve BP control in younger patients with hypertension, especially when combined with case management [27]. Recent publication of the Systolic Blood Pressure Intervention Trial, a study that demonstrated clinically important benefits to lowering SBP to ≈120 mmHg in high-risk individuals (excluding those with diabetes [10]), also supports the need for close monitoring [70]. Given these low BP targets, careful BP monitoring will be required to operationalize this intervention in current clinical practice.

A critical question is whether BP telemonitoring can be successfully implemented in seniors, who may be less technologically savvy than younger individuals. We propose to test a very simple system that does not require specialized expertise. Usability and acceptability testing constitute a central objective of the trial. Qualitative studies of younger patients and of care providers have, in general, shown that patients find BP telemonitoring usable and acceptable but that providers express concerns about workload, troubleshooting the technology, and increased need for resources [71,72]. This makes rigorous cost-effectiveness analysis essential. It also underscores the importance of minimizing monitoring to include only measurements that are clinically necessary.

Enrolment within the TECHNOMED trial is expected to begin in mid-2016. Recruitment of all 300 subjects is expected by mid-2018. Final results for the main study are anticipated by 2020. We anticipate that this trial will clarify the advantages and disadvantages of BP telemonitoring in this high-risk population with both diabetes and hypertension (see Multimedia Appendix 1).

## Appendix

Multimedia Appendix 1 CIHR Reviews.

## Abbreviations

ABPM: ambulatory blood pressure monitoring
BP: blood pressure
RCTs: randomized controlled trials
SBP: systolic blood pressure
TECHNOMED: Telemonitoring and Protocolized Case Management for Hypertensive Community-Dwelling Seniors With Diabetes
