# Supplementary material for: Telemonitoring and Protocolized Case Management for Hypertensive Community-Dwelling Seniors With Diabetes: Protocol of the TECHNOMED Randomized Controlled Trial
Source: JMIR Res Protoc. 2016 Jun 24;5(2):e107. doi: 10.2196/resprot.5775 (PMC4938881; doi:10.2196/resprot.5775)
Supplement: Multimedia Appendix 1 [file resprot_v5i2e107_app1.pdf]

Review Type/Type d'évaluation: Committee Member 1/Membre de comité 1
Name of Applicant/Nom du chercheur: PADWAL, Raj Singh
Application No./Numéro de demande: 348217
Agency/Agence: CIHR/IRSC
Competition/Concours: 2015-06-03 Operating Grant: eHealth Innovations Initiative: eHealth Innovation Partnership Program (eHIPP)/Subvention de fonctionnement: Initiative Innovations en cybersanté : Programme de partenariats pour l'innovation en cybersanté (PPIC)
Committee/Comité: eHealth Innovation Partnership Program (eHIPP): Support of Seniors/Programme de partenairiats pour l'innovation en cybersanté-L'appui awx personnes âgéess
Title/Titre: Telemonitoring and Protocolized Case Management for Hypertension In Seniors

---

Assessment/Évaluation:

Telemonitoring and Protocolozed Case Management for Hypertension in Seniors

Planned Intervention:

Relevance of the population and challenges, gaps, and inefficiencies to be addressed by e-Health enabled innovations: Very relevant population and condition with documented system inefficiencies to be addressed by the study.
Technology Readiness Level: No issues - pilot test of technology completed with 20 users.
Probability intervention will improve outcomes: Highly likely looking at past studies.

Probability the innovation enabled care delivery program will be more cost effective than usual care: US study suggests that this should be the case.
Potential scalability of the planned intervention: Easily scalable.

eHealth Innovation Evaluation:

Appropriateness of population and sample size: Convenience sample of residents in 43 multi-occupancy Greater Edmonton Foundation Seniors and Rosedale Seniors housing. Sample size recognizes potential attrition risk with this population and accounts for this to ensure adequate power.
Sample size: Need - 80 patients per arm = 240 total. Accounting for ≈20% attrition over 1 year will recruit 100 patients per arm = 300 total.
Quality of measurement of outcomes and interventions: The outcomes and measures appear well thought through and comprehensive, capturing qualitative and quantitative data that will provide rich evidence.

Appropriateness and quality of approach to bias control to optimize internal validity: Risk of confounding factors, including receiving case management from other parties, has been evaluated. The applicants recognize that the residents of the multi-occupancy units may discuss the study and argue that this should have minimal impact as home BP monitoring alone has minimal impact on BP control. Recent studies by Kloseck et al (currently under review) have demonstrated that this type of study in a retirement community can change personal behaviour and have an impact upon outcomes and those in the control arm did modify their behaviour and followed up individually with their clinicians. For this reason it is suggested that the applicants may wish to consider a second control group - one at some distance from those residents receiving the interventions, as a comparator to those control patients within the trial residences.

**Review Type/Type d'évaluation:**	Committee Member 1/Membre de comité 1
**Name of Applicant/Nom du chercheur:**	PADWAL, Raj Singh
**Application No./Numéro de demande:**	348217
**Agency/Agence:**	CIHR/IRSC
**Competition/Concours:**	2015-06-03 Operating Grant: eHealth Innovations Initiative: eHealth Innovation Partnership Program (eHIPP)/Subvention de fonctionnement: Initiative Innovations en cybersanté : Programme de partenariats pour l'innovation en cybersanté (PPIC)
**Committee/Comité:**	eHealth Innovation Partnership Program (eHIPP): Support of Seniors/Programme de partenairiats pour l'innovation en cybersanté-L'appui awx personnes âgéess
**Title/Titre:**	Telemonitoring and Protocolized Case Management for Hypertension In Seniors

Assessment/Évaluation:

Appropriateness and quality of approach to cost effectiveness evaluation: Detailed cost effectiveness evaluation methodology has been provided and seems appropriate.
Appropriateness and quality of approach to analysis and sex/gender analysis: Sex and other patient characteristics included in analysis.
Suitability of environment to conduct proposed activities: No issues: data collection, storage and access information provided and seems robust. Risk of data transmission recognized but is being undertaken by a company with experience in this.
Economic evaluation of complex health system interventions: Evaluation includes analysis at patient and system level recognizing that much of the effect may occur downstream.

Team:
The quality of the partnership between the health-care innovation community and the health technology partners and the likelihood it will succeed: Many of the research team members have clearly collaborated in the past (evidenced in peer reviewed articles and successful grant applications) and the development of the prototype is evidence of working with the technology provider.
The capacity of the scientific team to undertake high quality evaluation using past experience and success: This seems to be a strong team with prior experience that will stand them in good stead to complete the RCT.

The quality of scientific, clinical, patient/end-user, and management leadership: This seems like a strong team but the addition of a KT specialist is recommended to assist in the dissemination and non-academic KTA activities.
Suitability of the environment to conduct proposed activities: No major concerns - the use of the multi-residence homes as a gateway to recruit, seems a logical way to proceed that should eliminate many of the barriers encountered by other studies.
Availability and accessibility of personnel, facilities and infrastructure required to conduct proposed activities: No concerns - plans seem to be in place for the required resources to undertake this study.

Budget:
Appropriateness of the budget to support proposed activities: While the costs are all clearly identified an attributed to the correct year, there are a number of expendables, services and travel costs that have 'other

**Review Type/Type d'évaluation:**	Committee Member 1/Membre de comité 1
**Name of Applicant/Nom du chercheur:**	PADWAL, Raj Singh
**Application No./Numéro de demande:**	348217
**Agency/Agence:**	CIHR/IRSC
**Competition/Concours:**	2015-06-03 Operating Grant: eHealth Innovations Initiative: eHealth Innovation Partnership Program (eHIPP)/Subvention de fonctionnement: Initiative Innovations en cybersanté : Programme de partenariats pour l'innovation en cybersanté (PPIC)
**Committee/Comité:**	eHealth Innovation Partnership Program (eHIPP): Support of Seniors/Programme de partenairiats pour l'innovation en cybersanté-L'appui awx personnes âgéess
**Title/Titre:**	Telemonitoring and Protocolized Case Management for Hypertension In Seniors

Assessment/Évaluation:

cash funding sources' identified but the text does not explain who/how these are being paid for. Further detail would be useful in this section, especially where costs for some years are requested from CIHR and for other years are provided by 'others'. All KTA is currently of the traditional academic form and it is suggested that some should be targeted for service providers and participants of the study. There could also be consideration of more 'within study' KTA rather than the current heavy end weighting.

Review Type/Type d'évaluation: Committee Member 2/Membre de comité 2
Name of Applicant/Nom du chercheur: PADWAL, Raj Singh
Application No./Numéro de demande: 348217
Agency/Agence: CIHR/IRSC
Competition/Concours: 2015-06-03 Operating Grant: eHealth Innovations Initiative: eHealth Innovation Partnership Program (eHIPP)/Subvention de fonctionnement: Initiative Innovations en cybersanté : Programme de partenariats pour l’innovation en cybersanté (PPIC)
Committee/Comité: eHealth Innovation Partnership Program (eHIPP): Support of Seniors/Programme de partenairiats pour l'innovation en cybersanté-L'appui awx personnes âgéess
Title/Titre: Telemonitoring and Protocolized Case Management for Hypertension In Seniors

Assessment/Évaluation:

The objective of this application is to examine the incremental effectiveness, safety, cost-effectiveness, usability, and acceptability of home blood pressure (HBP) telemonitoring used with or without protocolized case management, compared to 'usual care' in community-dwelling seniors with diabetes and hypertension. Consenting patients from two housing complexes will be be randomized to 'usual care', home BP telemonitoring alone and home BP telemonitoring plus protocolized pharmacist case management. In the telemonitored arms, providers and patients will receive/access telemonitored BP data summaries. Usual care patients will receive a home BP monitor only but neither they nor their providers will access functional, usable teletransmitted data. Key outcome measures will be collected at 6 and 12 months.

Planned Intervention:
The application clearly articulates the population, challenges and gaps faced in managing current BP therapeutic ranges and the impact the ehealth innovation can potentially have in improving management and patient outcomes. The readiness level of the technology is 6-7 and a prototype of the technology has been developed and now needs to be integrated into the health technology partner's platform (TeleMED). Previous research has indicated that BP telemonitoring improves patient outcomes but this has not been tested in the older adult population which is the purpose of this application. The proposed study will test HBP telemonitoring in the community-dwelling older adult population and it already discusses cost savings from other studies. The proposed ehealth solution will be cost-effective if it is easier for seniors to use and to report readings. End-users are included on the team and a patient-centered focus exists in terms of checking usability and acceptability of the ehealth innovation. The change management and implementation plan involves team members with multiple roles who therefore have the ability to influence change management at various levels. The ehealth innovation can be scaled up as platforms already exist with TeleMED for ABPM and so can incorporate HBP monitoring as well. The approach to monitoring legal and ethical issues is sound and it is possible to do with the current systems already in place and used by TeleMED and EMR. The value of the ehealth innovation for clinicians is high as it would help to monitor patients and flag if urgent. The innovation also has high value for patients in terms of knowing when to see their physician.

eHealth Innovation Evaluation:
Highly relevant outcomes are proposed to be measured: patients' QoL, satisfaction, intervention usability and acceptability; clinician - view trends and changes in BP management and that it is within the therapeutic range; health care system - cost-effectiveness and outcomes achieved. The applicants propose to conduct a

**Review Type/Type d'évaluation:**	Committee Member 2/Membre de comité 2
**Name of Applicant/Nom du chercheur:**	PADWAL, Raj Singh
**Application No./Numéro de demande:**	348217
**Agency/Agence:**	CIHR/IRSC
**Competition/Concours:**	2015-06-03 Operating Grant: eHealth Innovations Initiative: eHealth Innovation Partnership Program (eHIPP)/Subvention de fonctionnement: Initiative Innovations en cybersanté : Programme de partenariats pour l'innovation en cybersanté (PPIC)
**Committee/Comité:**	eHealth Innovation Partnership Program (eHIPP): Support of Seniors/Programme de partenairiats pour l'innovation en cybersanté-L'appui awx personnes âgéess
**Title/Titre:**	Telemonitoring and Protocolized Case Management for Hypertension In Seniors

Assessment/Évaluation:

usability and acceptability test prior to undertaking the RCT and again at the end of the study period. The applicants also propose to ask end users to take BP measurements and access the web portal and describe verbally what they are doing while they provide one-to-one feedback to the researchers. This approach enables the researchers to observe how study participants use the technology. In addition they will also use likert scales to rate the device, teletransmission, health portal etc. The environment suggested for conducting the study is suitable with commitment from two seniors housing complexes.

Team:

The partnership with the technology partners is strong. TeleMED already has ABPM and adding HBPM would increase business. The prototype has already been developed by the research team and needs to be incorporated into the TeleMED platform with refinements. The other partner (Pharmacare) already provides case management in seniors' buildings so could expand their business as well. The capacity to integrate the ehealth solution is high with senior leadership on board with multiple roles and past experience in testing the usability and acceptability. In addition the research team has developed the prototype and pilot testing. Experts with tech-assisted care will work with TeleMED to incorporate the prototype into the TeleMED platform so the infrastructure exists.

Review Type/Type d'évaluation: Committee Member 3/Membre de comité 3
Name of Applicant/Nom du chercheur: PADWAL, Raj Singh
Application No./Numéro de demande: 348217
Agency/Agence: CIHR/IRSC
Competition/Concours: 2015-06-03 Operating Grant: eHealth Innovations Initiative: eHealth Innovation Partnership Program (eHIPP)/Subvention de fonctionnement: Initiative Innovations en cybersanté : Programme de partenariats pour l'innovation en cybersanté (PPIC)
Committee/Comité: eHealth Innovation Partnership Program (eHIPP): Support of Seniors/Programme de partenairiats pour l'innovation en cybersanté-L'appui awx personnes âgéess
Title/Titre: Telemonitoring and Protocolized Case Management for Hypertension In Seniors

---

Assessment/Évaluation:

Innovation - positive

Once technology developed there will be little cost to patients who already have smart phones

Pharmacy integration is a novel and useful appliation

Negative

Relies heavily onfamily and/or care providers. Can help with reminders and consistent motivation for consistent participation

H-Health

Telemed partner seems to already be ingrained and has developed Vitel Flo

**Review Type/Type d'évaluation:**	Committee Member 3/Membre de comité 3
**Name of Applicant/Nom du chercheur:**	PADWAL, Raj Singh
**Application No./Numéro de demande:**	348217
**Agency/Agence:**	CIHR/IRSC
**Competition/Concours:**	2015-06-03 Operating Grant: eHealth Innovations Initiative: eHealth Innovation Partnership Program (eHIPP)/Subvention de fonctionnement: Initiative Innovations en cybersanté : Programme de partenariats pour l’innovation en cybersanté (PPIC)
**Committee/Comité:**	eHealth Innovation Partnership Program (eHIPP): Support of Seniors/Programme de partenairiats pour l'innovation en cybersanté-L'appui awx personnes âgéess
**Title/Titre:**	Telemonitoring and Protocolized Case Management for Hypertension In Seniors

Assessment/Évaluation:

Patient partners?

Review Type/Type d'évaluation: Committee Member 4/Membre de comité 4
Name of Applicant/Nom du chercheur: PADWAL, Raj Singh
Application No./Numéro de demande: 348217
Agency/Agence: CIHR/IRSC
Competition/Concours: 2015-06-03 Operating Grant: eHealth Innovations Initiative: eHealth Innovation Partnership Program (eHIPP)/Subvention de fonctionnement: Initiative Innovations en cybersanté : Programme de partenariats pour l'innovation en cybersanté (PPIC)
Committee/Comité: eHealth Innovation Partnership Program (eHIPP): Support of Seniors/Programme de partenairiats pour l'innovation en cybersanté-L'appui awx personnes âgéess
Title/Titre: Telemonitoring and Protocolized Case Management for Hypertension In Seniors

---

Assessment/Évaluation:

A large percentage of people have incorrect blood pressure and also suffer from other conditions such as diabetes. Of these people do not seek medical help or provide information to health professionals because they don't know they need to, they want to remain in the comfort of their home or the effort required to seek assistance. Two team members have developed a blood pressure monitor that resides with the patient at home and connects via Bluetooth to a smart phone allowing the transmission of readings to a web portal where they can be viewed by health professionals.

While a large demographic do have high blood pressure and suffer from diabetes, this technology could also be expanded to other demographics that have issues such as rural patients or aboriginal communities. The large factor in this technology meeting the TRL requirements is the use of off the shelf technology and having a working prototype. It is critical to keep a solution like this very simple for the patient. Adoption by patients is key and if it is complex or hard to use it will simply collect dust. Given the high percentage of people who do not currently report data this solution has the ability of lead to better patient outcomes. While the server sits behind a firewall with only a single port, some additional consideration should be given to things such as "port knocking". Passwords should not be stored in plain text and should be salted and properly encrypted. For clinicians logging in to the portal, two factor authentication should be considered for an added level of security.

The team has a strong group with expertise on the relevant population and in particular, in-house expertise on technology which will allow for better working and technology integration between the health professionals and the technology partner. The partners are good stake holders and include senior management from academia, national associations and people who have been doing health and technology for a while and have internal interests in seeing this succeed and continue beyond.

The partners individually are well established with a proven track record offering services to a much larger population. These are not "startups" and have the capacity to commercialize the technology if this is a success. The technology itself is not ground breaking and uses off the shelf components but that is a strength and will increase the chances of a successful rollout. The environment for this project is well defined and assuming the final solution has good usability by the patient there is a high adoption potential. This could save the health system money, provide a better patient experience and give health professionals more information sooner.

**Review Type/Type d'évaluation:**	Committee Member 4/Membre de comité 4
**Name of Applicant/Nom du chercheur:**	PADWAL, Raj Singh
**Application No./Numéro de demande:**	348217
**Agency/Agence:**	CIHR/IRSC
**Competition/Concours:**	2015-06-03 Operating Grant: eHealth Innovations Initiative: eHealth Innovation Partnership Program (eHIPP)/Subvention de fonctionnement: Initiative Innovations en cybersanté : Programme de partenariats pour l'innovation en cybersanté (PPIC)
**Committee/Comité:**	eHealth Innovation Partnership Program (eHIPP): Support of Seniors/Programme de partenairiats pour l'innovation en cybersanté-L'appui awx personnes âgéess
**Title/Titre:**	Telemonitoring and Protocolized Case Management for Hypertension In Seniors

Assessment/Évaluation:

In general the budget is acceptable that being said some other considerations should be taken towards the Android technology options. The Android platform is the current choice and a good one given its openness and flexibility. With this in mind, applicants should consider cheaper alternative Android phones. Given the choice of a low end model of Samsung phone and the low hardware requirements (state of the art phone not required), a cheaper alternative should be considered. For example, something like a Lenovo A1900 Android phone (purchased online and not from a carrier) is approximately one third of the cost with similar specifications to the Samsung and still being operational on the Rogers phone network as per the quote. Purchasing this in bulk would reduce the cost more and would be less likely to be stolen and cheaper to replace in the event of damage.

It is recommended to be funded with consideration given to cheaper off the shelf mobile phone hardware.
